# Supplementary material for: Acquisition of oral microbiota is driven by environment, not host genetics
Source: Microbiome. 2021 Feb 23;9:54. doi: 10.1186/s40168-020-00986-8 (PMC7903647; doi:10.1186/s40168-020-00986-8)
Supplement: Supplementary file 2 — Additional file 1: Supplementary Figure S1. Strain-level community characterization led to increased separation between samples. Comparison of centroid distances for strain and species level communities, both in terms of subject type (mother/child) separation and sampling site (saliva/swab, subgingival and supragingival plaque). Samples were significantly better separated at the strain level. P-values were generated using paired Wilcoxon rank sum test (significance level *** refers to p < 0.001). Supplementary Figure S2. Beta-diversity comparison among samples by subject and family types, at both species and strain levels. Non-metric multidimensional scaling (NMDS) plots using Bray-Curtis dissimilarities based on community membership, at ISRstrain level (top) and 16S Species level (bottom) are shown. Figure S3. No influence of genetics on sharing of strains between mother and child using Jaccard dissimilarities. The saliva/soft tissue swab samples were also analyzed using the Jaccard dissimilarity indices computed based on presence/absence of ISR strains. The results were very similar to what was obtained using Bray-Curtis dissimilarities. No significant difference was observed in the mother-child dissimilarities between the biologic and adoptive groups, and both biologic and adoptive children’s oral microbiota were significantly more similar to their own mothers than unrelated mothers. Distribution of distances are shown using violin plots, with embedded box and whisker plots. Biological vs adoptive statistical comparisons were performed using Wilcoxon rank sum test, and related/unrelated comparisons were performed using the previously described permutation test. Figure S4. No influence of genetics on sharing of strains between mother and child using relative abundance of strains. Bray-Curtis dissimilarities between the mother-child pairs for the saliva/soft tissue swab samples were also computed based on relative abundance of ISR strains. No significant difference [file 40168_2020_986_MOESM2_ESM.pdf]

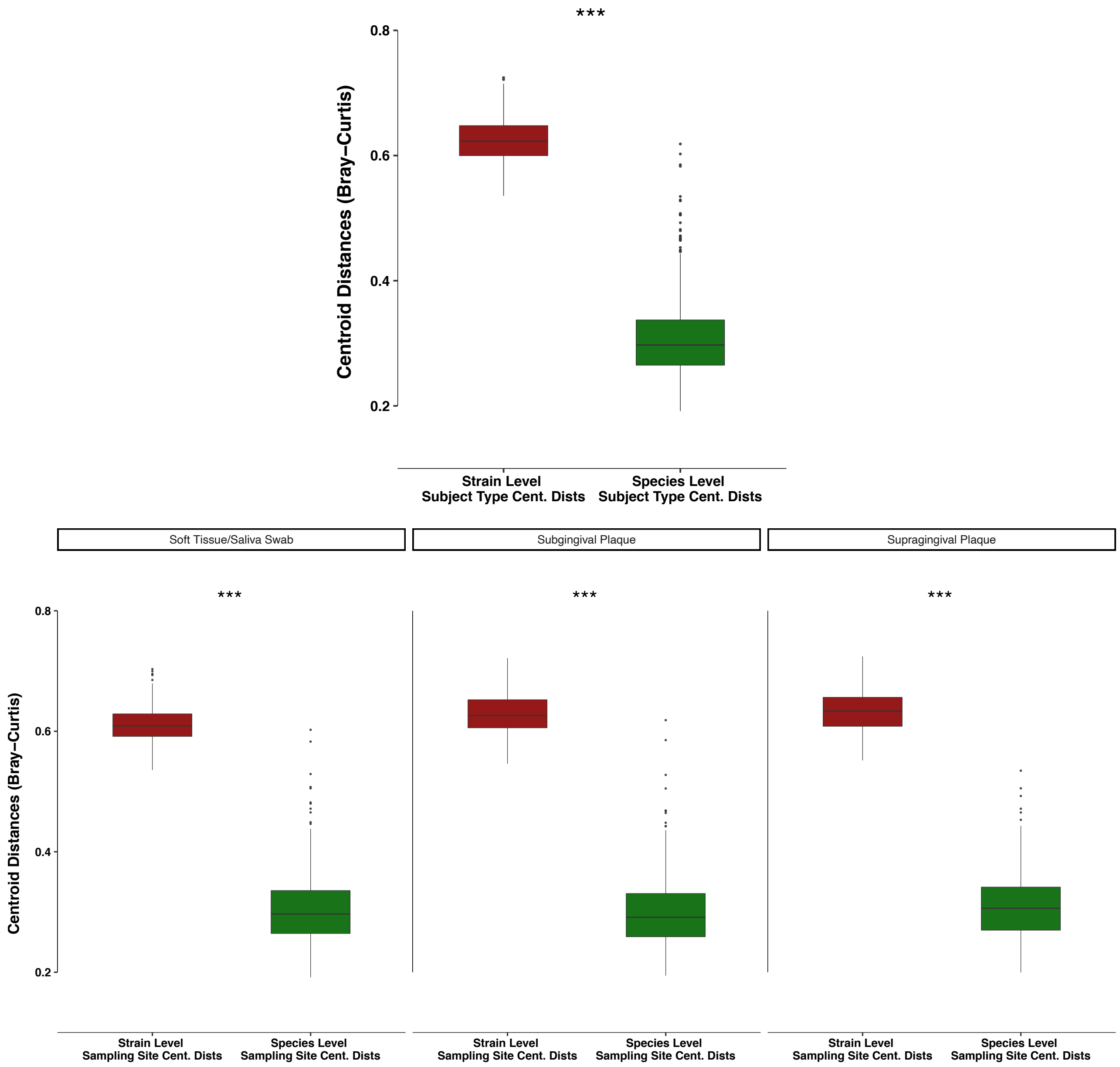

Supplementary Figure S1. Strain-level community characterization led to increased separation between samples. Comparison of centroid distances for strain and species level communities, both in terms of subject type (mother/child) separation and sampling site (saliva/swab, subgingival and supragingival plaque). Samples were significantly better separated at the strain level. P-values were generated using paired Wilcoxon rank sum test (significance level *** refers to $p < 0.001$).

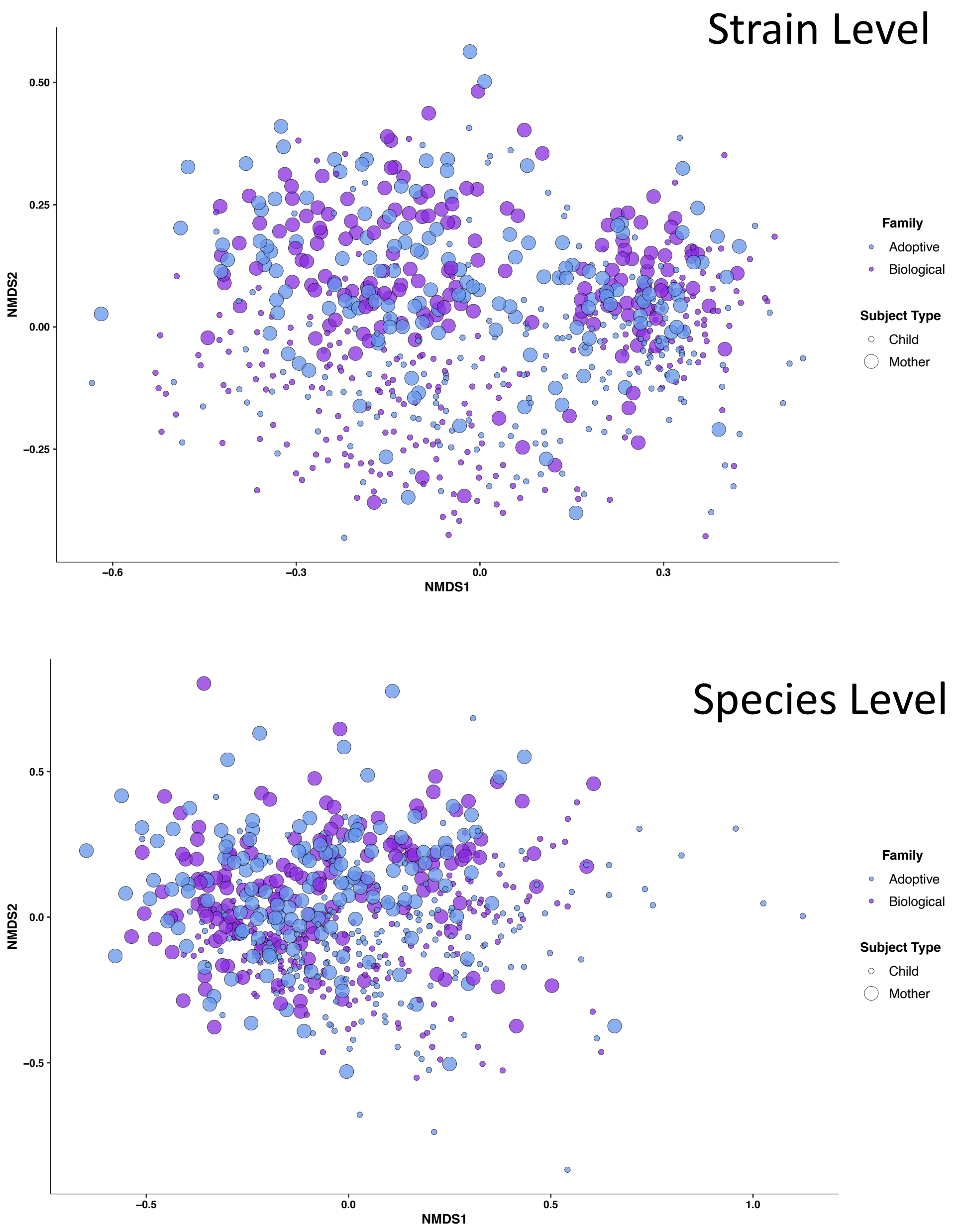

Supplementary Figure S2. Beta-diversity comparison among samples by subject and family types, at both species and strain levels. Non-metric multidimensional scaling (NMDS) plots using Bray-Curtis dissimilarities based on community membership, at ISR-strain level (top) and 16S Species level (bottom) are shown.

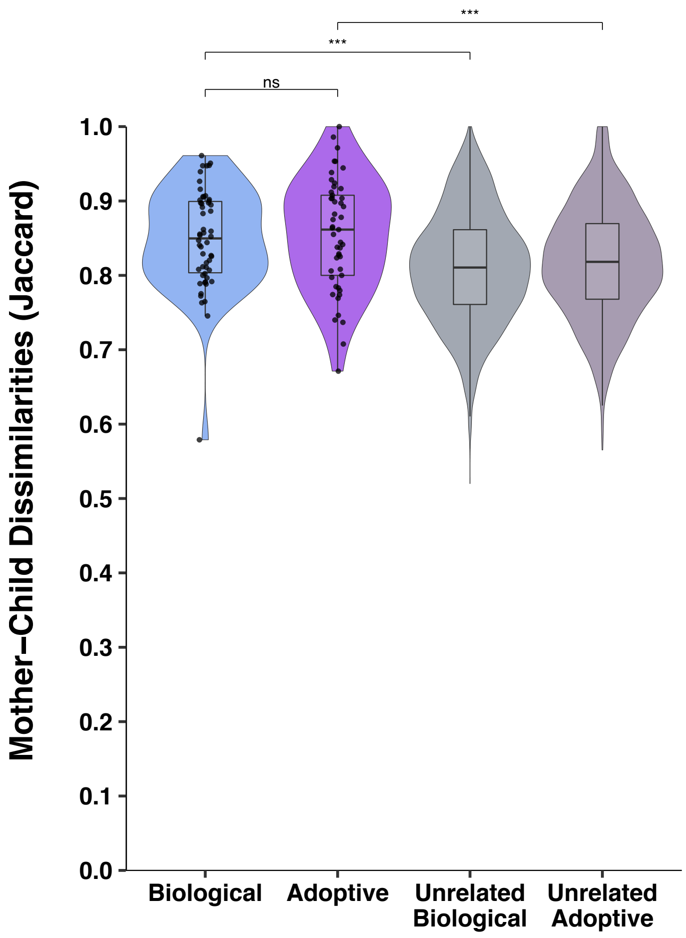

Figure S3. No influence of genetics on sharing of strains between mother and child using Jaccard dissimilarities. The saliva/soft tissue swab samples were also analyzed using the **Jaccard dissimilarity** indices computed based on presence/absence of ISR strains. The results were very similar to what was obtained using Bray-Curtis dissimilarities. No significant difference was observed in the mother-child dissimilarities between the biologic and adoptive groups, and both biologic and adoptive children's oral microbiota were significantly more similar to their own mothers than unrelated mothers. Distribution of distances are shown using violin plots, with embedded box and whisker plots. Biological vs adoptive statistical comparisons were performed using Wilcoxon rank sum test, and related/unrelated comparisons were performed using the previously described permutation test.

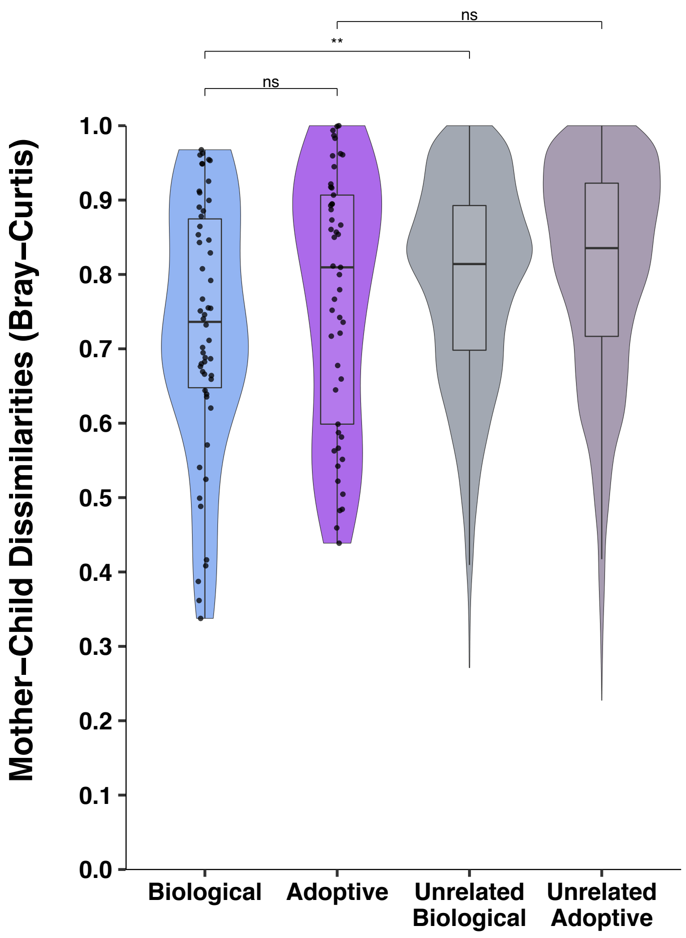

Figure S4. No influence of genetics on sharing of strains between mother and child using relative abundance of strains. Bray-Curtis dissimilarities between the mother-child pairs for the saliva/soft tissue swab samples were also computed based on **relative abundance of ISR strains**. No significant difference was observed in the mother-child dissimilarities between the biologic and adoptive groups. While the biological group children's oral microbiota was significantly more similar to their own mothers than unrelated mothers, the same distinction could not be made for the adoptive group. Biological vs adoptive statistical comparisons were performed using Wilcoxon rank sum test, and related/unrelated comparisons were performed using the previously described permutation test.

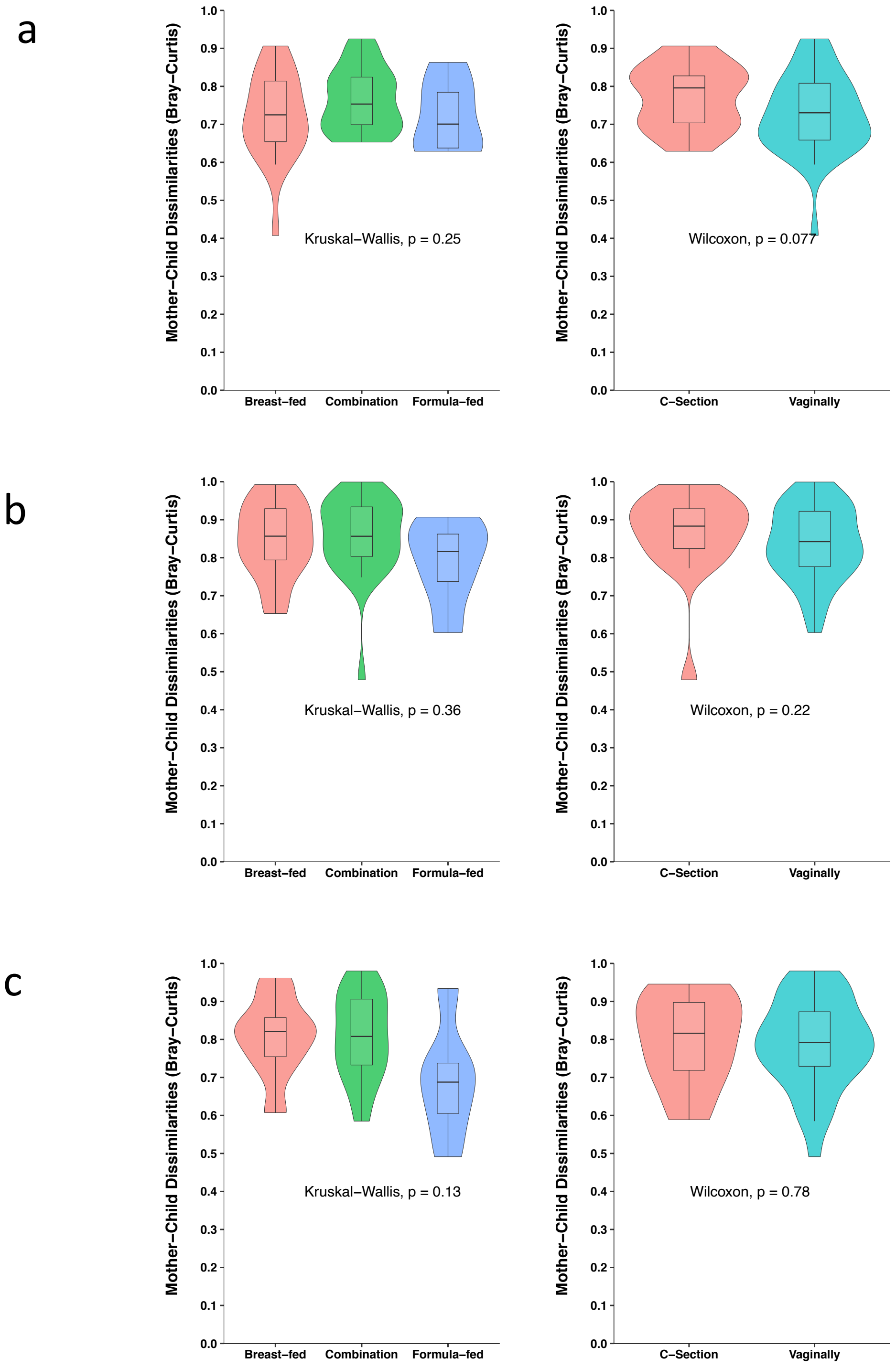

Figure S5. Effect of feeding and delivery modes on mother-child distances. Differences in feeding mode (right) or delivery mode (left) among the biological group children did not have any significant effect on the mother-child dissimilarities, for either the **a)** saliva/soft tissue swab, **b)** supragingival or **c)** subgingival plaque samples.

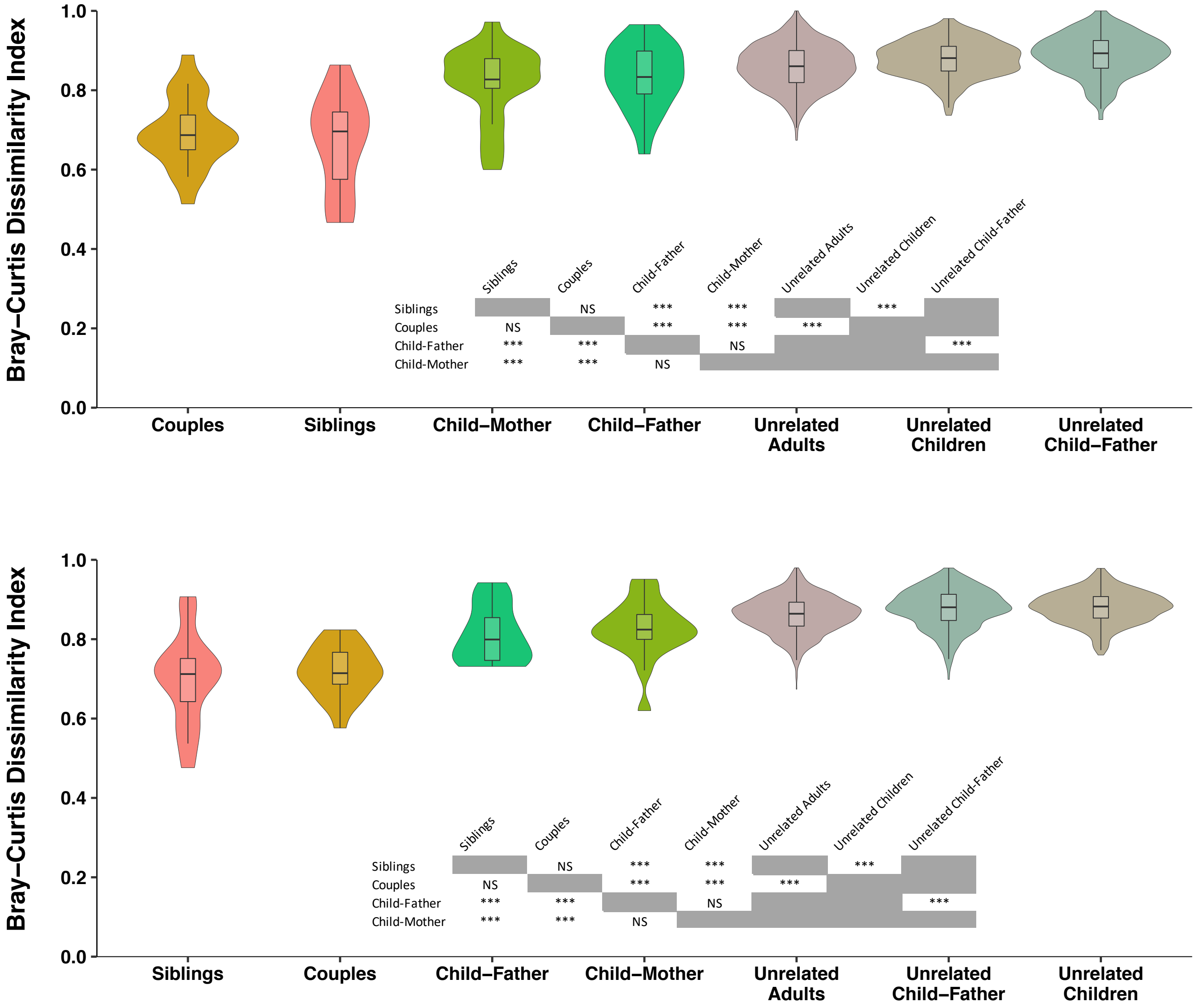

Figure S6. Extended family comparisons using plaque samples show results similar to saliva samples. Comparing microbial community similarities among different family groups, based on **supragingival plaque** (**top**) and **subgingival plaque** (**bottom**) samples from the extended biological family dataset. Groupings are ordered based on increasing median distances. Shared environment/contact lead to greater oral microbiota composition similarity, and no evidence of genetic influence was detected. Statistical comparisons were performed using Wilcoxon rank sum test and a custom permutation test (when including unrelated groups).

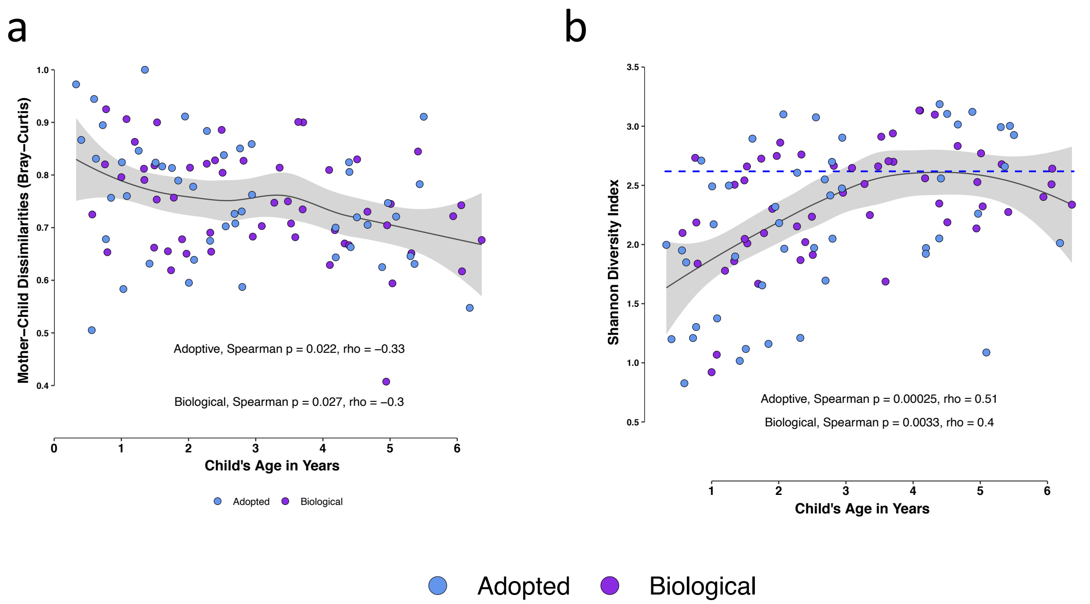

Figure S7. Relationship of child's age with mother-child dissimilarities or alpha diversity was not different for adoptive or biological group children.

a. Scatterplot exhibiting the relationship of mother-child distances with age of the child.

b. Plot for Shannon Diversity Index versus child's age. The blue dotted line represents mean Shannon Diversity for mothers of those children.

Strength and direction of associations were measured using Spearman's rank-order test. Scatter plots were smoothed using the regression method LOESS fit. Statistics were computed separately for the adoptive and biological group children, and two older children (>=10 years) were excluded. Analysis was based on strain level communities.

Group	Subject	Site	Samples
Adoptive Families	Child	Saliva/Soft Tissue	60
		Subgingival	58
		Supragingival	60
	Mother	Saliva/Soft Tissue	50
		Subgingival	48
		Supragingival	49
	Father	Saliva/Soft Tissue	1
		Subgingival	1
		Supragingival	1
Biological Families	Child	Saliva/Soft Tissue	74
		Subgingival	73
		Supragingival	74
	Mother	Saliva/Soft Tissue	55
		Subgingival	54
		Supragingival	55
	Father	Saliva/Soft Tissue	22
		Subgingival	21
		Supragingival	22
**Total ISR Samples:**			778

Supplementary Table ST1. Details of number of samples for which sequencing data was available in each group for the ISR dataset.

Group	Subject	Site	Samples
Adoptive Families	Child	Saliva/Soft Tissue	59
		Subgingival	56
		Supragingival	59
	Mother	Saliva/Soft Tissue	49
		Subgingival	47
		Supragingival	50
	Father	Saliva/Soft Tissue	1
		Subgingival	1
		Supragingival	1
Biological Families	Child	Saliva/Soft Tissue	55
		Subgingival	54
		Supragingival	56
	Mother	Saliva/Soft Tissue	54
		Subgingival	52
		Supragingival	54
	Father	Saliva/Soft Tissue	22
		Subgingival	18
		Supragingival	21
**Total 16S Samples:**			709

Supplementary Table ST2. Details of number of samples for which sequencing data was available in each group for the 16S dataset.

Species OTUs	Biological Families		Adoptive Families		Combined	
	Shared %	Abundance %	Shared %	Abundance %	Shared %	Abundance %
Granulicatella adiacens	100	2	100	1.9	100	2.0
Haemophilus parainfluenzae	100	5.2	100	3.5	100	4.4
Prevotella melaninogenica	100	6.3	97.8	4.7	99	5.5
Rothia mucilaginosa	100	15.1	100	14.1	100	14.6
Streptococcus mitis pneumoniae infantis oralis	100	24.2	100	25.4	100	24.8
Veillonella atypica dispar parvula	100	7.9	100	7.5	100	7.7
Actinomyces odontolyticus lingnae	97.9	0.9	93.3	0.9	96	0.9
Porphyromonas CW034	93.8	1.5	86.7	1.5	90	1.5
Rothia dentocariosa	93.8	0.5	91.1	1.3	92	0.9
Streptococcus australis	93.8	1.3	93.3	0.9	94	1.1
Streptococcus parasanguinis	93.8	3.2	91.1	3.2	92	3.2
Gemella sanguinis	91.7	0.4	93.3	0.5	92	0.4
Oribacterium sinus	91.7	0.6	93.3	0.6	92	0.6
Gemella haemolysans	89.6	0.4	95.6	1.1	93	0.7
Lachnoanaerobaculum umeaense	89.6	0.2	84.4	0.2	87	0.2
Neisseria flavescens	89.6	4.8	86.7	4	88	4.4
Streptococcus vestibularis salivarius	89.6	5	95.6	5.1	93	5.0
Streptococcus VG051	89.6	2.8	93.3	1.9	91	2.4
Campylobacter concisus	87.5	0.3	82.2	0.3	85	0.3
Fusobacterium periodonticum	87.5	1.5	82.2	1.5	85	1.5
Leptotrichia BU064	87.5	0.5	88.9	0.5	88	0.5
Streptococcus cristatus	87.5	0.8	80	1.2	84	1.0
Streptococcus sanguinis	87.5	0.6	82.2	0.9	85	0.7
Neisseria meningitidis polysaccharea	85.4	1.4	80	0.5	83	1.0

Supplementary Table ST3. List of the most widely shared species among the mother-child pairs in each group, along with their relative abundance in the dataset. Data is based on saliva/soft tissue swab samples.

Species OTU	# of ISR-type Strains	Total Sequence Counts	% of Subjects
Streptococcus mitis pneumoniae infantis oralis	183	3648025	100
Rothia mucilaginosa	203	1323837	98.95
Streptococcus vestibularis salivarius	32	364929	95.44
Streptococcus parasanguinis	85	364200	95.44
Granulicatella adiacens	200	307273	98.95
Streptococcus australis	11	166784	83.16
Streptococcus sanguinis	44	115959	82.46
Gemella sanguinis	30	95432	88.77
Atopobium parvulum	9	52451	72.98
Veillonella atypica dispar parvula	103	43473	83.51
Neisseria meningitidis polysaccharea	13	42371	59.3
Moraxella osloensis	116	38833	46.67
Streptococcus gordonii	16	39042	35.79
Haemophilus parainfluenzae	43	31421	68.77
Gemella haemolysans	46	26062	48.07
Streptococcus cristatus	14	16893	43.86
Gemella morbillorum	21	15446	30.53
Rothia aeria	28	14582	46.32
Rothia dentocariosa	28	9174	19.65
Streptococcus intermedius constellatus	10	7077	16.84

Supplementary Table ST4. List of the 20 most abundant oral bacteria species. Data is based on saliva/soft tissue swab samples.
