# Supplementary material for: Acquisition of oral microbiota is driven by environment, not host genetics
Source: Microbiome. 2021 Feb 23;9:54. doi: 10.1186/s40168-020-00986-8 (PMC7903647; doi:10.1186/s40168-020-00986-8)
Supplement: Supplementary file 3 — Additional file 2: Supplementary Table ST5. Summary statistics table. [file 40168_2020_986_MOESM3_ESM.docx]

|  | |  |  |  |  |
| --- | --- | --- | --- | --- | --- |
| **Variable** | **level** | | **Adopted** | **Biological** | **p** |
| n |  | | 49 | 54 |  |
| Age (mean (SD)) |  | | 2.88 (2.10) | 3.20 (1.87) | 0.415 |
| Gender (%) | Female | | 17 ( 34.7) | 29 (53.7) | 0.082 |
|  | Male | | 32 ( 65.3) | 25 (46.3) |  |
| Delivery Mode (%) | C-Section | | 12 ( 26.7) | 19 (35.2) | 0.489 |
|  | Vaginally | | 33 ( 73.3) | 35 (64.8) |  |
| Feeding Mode (%) | Breast-fed | | 0 ( 0.0) | 21 (38.9) | <0.001 |
|  | Combination | | 1 ( 2.1) | 27 (50.0) |  |
|  | Formula-fed | | 46 ( 97.9) | 6 (11.1) |  |
| Race (%) | Black or African American | | 17 ( 34.7) | 1 ( 1.9) | <0.001 |
|  | Mixed | | 8 ( 16.3) | 5 ( 9.3) |  |
|  | White | | 24 ( 49.0) | 48 (88.9) |  |
| Plaque level (%) | Mild | | 17 ( 37.8) | 27 (51.9) | 0.321 |
|  | Moderate | | 3 ( 6.7) | 4 ( 7.7) |  |
|  | None | | 25 ( 55.6) | 21 (40.4) |  |
| Tongue Biofilm (%) | Heavy | | 1 ( 2.2) | 0 ( 0.0) | <0.001 |
|  | Light | | 21 ( 45.7) | 6 (11.5) |  |
|  | None | | 24 ( 52.2) | 46 (88.5) |  |
| Gingivitis (%) | Mild | | 11 ( 23.9) | 25 (48.1) | 0.045 |
|  | Moderate | | 2 ( 4.3) | 2 ( 3.8) |  |
|  | None | | 33 ( 71.7) | 25 (48.1) |  |
| School (%) | Daycare | | 18 ( 36.7) | 24 (44.4) | 0.305 |
|  | kindergarten | | 2 ( 4.1) | 4 ( 7.4) |  |
|  | MD | | 0 ( 0.0) | 3 ( 5.6) |  |
|  | None | | 20 ( 40.8) | 15 (27.8) |  |
|  | Pre School | | 8 ( 16.3) | 8 (14.8) |  |
|  | School | | 1 ( 2.0) | 0 ( 0.0) |  |
| Caries Level (%) | 0 | | 42 ( 85.7) | 46 (85.2) | 0.639 |
|  | 1 | | 1 ( 2.0) | 2 ( 3.7) |  |
|  | 2 | | 2 ( 4.1) | 4 ( 7.4) |  |
|  | 4 | | 1 ( 2.0) | 2 ( 3.7) |  |
|  | 5 | | 1 ( 2.0) | 0 ( 0.0) |  |
|  | 8 | | 1 ( 2.0) | 0 ( 0.0) |  |
|  | 11 | | 1 ( 2.0) | 0 ( 0.0) |  |
| Antibiotics Past Month (%) | No | | 47 ( 95.9) | 49 (90.7) | 0.515 |
|  | Yes | | 2 ( 4.1) | 5 ( 9.3) |  |
| Mother’s Caries (%) | No | | 6 ( 12.2) | 16 (29.6) | 0.056 |
|  | Yes | | 43 ( 87.8) | 38 (70.4) |  |
| Mother’s Gingivitis (%) | Mild | | 25 ( 52.1) | 14 (26.4) | 0.008 |
|  | Moderate | | 7 ( 14.6) | 5 ( 9.4) |  |
|  | None | | 16 ( 33.3) | 34 (64.2) |  |
| Mother’s Plaque Level (%) | Mild | | 34 ( 70.8) | 23 (43.4) | 0.002 |
|  | Moderate | | 5 ( 10.4) | 2 ( 3.8) |  |
|  | None | | 9 ( 18.8) | 28 (52.8) |  |
| Mother’s Ethnicity (%) | Hispanic or Latino | | 0 ( 0.0) | 2 ( 3.7) | 0.519 |
|  | Non Hispanic or Latino | | 49 (100.0) | 52 (96.3) |  |
| Mother’s tongue biofilm (%) | Heavy | | 1 ( 2.1) | 1 ( 1.9) | 0.004 |
|  | Light | | 32 ( 66.7) | 18 (34.0) |  |
|  | None | | 15 ( 31.2) | 34 (64.2) |  |
| Mother’s antibiotics (%) | No | | 47 ( 95.9) | 45 (84.9) | 0.125 |
|  | Yes | | 2 ( 4.1) | 8 (15.1) |  |
| Mother’s race (%) | Asian | | 0 ( 0.0) | 3 ( 5.6) | 0.246 |
|  | Black or African American | | 1 ( 2.0) | 1 ( 1.9) |  |
|  | White | | 48 ( 98.0) | 50 (92.6) |  |
| Caries yes/no (%) | No | | 42 ( 85.7) | 46 (85.2) | 1.000 |
|  | Yes | | 7 ( 14.3) | 8 (14.8) |  |
| Daycare (%) | No | | 20 ( 40.8) | 15 (29.4) | 0.324 |
|  | Yes | | 29 ( 59.2) | 36 (70.6) |  |
| Mother’s Age (mean (SD)) |  | | 40.19 (6.12) | 34.09 (4.34) | <0.001 |
| Mother-child race match (%) | No | | 24 ( 49.0) | 5 ( 9.3) | <0.001 |
|  | Yes | | 25 ( 51.0) | 49 (90.7) |  |

**Supplementary Table ST5. Summary statistics table.**
